# Head‐to‐head comparison of [^18^F]SMBT‐1 and [^18^F]DPA‐714 autoradiography on postmortem human brain samples

**DOI:** 10.1002/alz70856_107356

**Published:** 2026-01-09

**Authors:** Ryuichi Harada, Kaede Kudo, Aiko Ishiki, Akio Akagi, Hiroaki Miyahara, Yasushi Iwasaki, Manabu Tashiro, Shozo Furumoto, Nobuyuki Okamura

**Affiliations:** ^1^ Tohoku Medical and Pharmaceutical University, Sendai, Miyagi, Japan; ^2^ Research Center for Accelerator and Radioisotope Science, Tohoku University, Sendai, Miyagi, Japan; ^3^ Institute of Development, Aging and Cancer, Tohoku University, Sendai city, Japan; ^4^ Institute of Development, Aging and Cancer, Tohoku University, Sendai, Miyagi, Japan; ^5^ Institute of Development, Aging and Cancer, Tohoku University, Sendai, Japan; ^6^ Aichi Medical University, Nagakute, Aichi, Japan; ^7^ Tohoku Medical and Pharmaceutical University, Sendai, Japan

## Abstract

**Background:**

Neuroinflammation is characterized by activated microglia and reactive astrocytes, which contribute to neurodegeneration in various neurological conditions such as Alzheimer's disease (AD). Imaging biomarkers, such as translocator protein 18kDa (TSPO) and monoamine oxidase‐B (MAO‐B), have been proposed and actively investigated in clinical research. The aim of this study was to compare the binding distribution of a MAO‐B PET tracer [^18^F]SMBT‐1 and a TSPO PET tracer [^18^F]DPA‐714 using postmortem human brain samples to better understand the PET imaging results.

**Method:**

*In vitro* autoradiography of [^18^F]SMBT‐1 (MAO‐B), [^18^F]DPA‐714 (TSPO), [^18^F]NAV4694 (Aβ), and [^18^F]MK‐6240 (Tau) was performed using postmortem human brain sections from control, AD, and Progressive supranuclear palsy patients. Immunohistochemical analysis of MAO‐B, GFAP, TSPO, and CD68 was performed to interpret the autoradiographic signals. Histochemical staining was performed using Cyano‐PiB.

**Result:**

Higher specific binding of [^18^F]SMBT‐1 and [^18^F]DPA‐714 was observed in Aβ‐positive/tau‐positive AD brain sections than in the control. However, the regional distribution of [^18^F]SMBT‐1 was significantly different from that of [^18^F]DPA‐714. [^18^F]SMBT‐1 showed patchy signals that were partially colocalized with that of Aβ detected by [^18^F]NAV4694. In contrast, [^18^F]DPA‐714 showed laminar distribution, which was not consistent with Aβ and tau pathology. Histochemical staining confirmed that MAO‐B‐positive astrocytes (GFAP‐positive) were surrounded by Cyano‐PiB‐positive Aβ plaques, even in Aβ‐positive/Tau‐negative control brain sections.

**Conclusion:**

[^18^F]SMBT‐1 detected MAO‐B‐positive reactive astrogliosis associated with Aβ and tau pathology in the AD continuum, whereas [^18^F]DPA‐714 detected TSPO‐positive cells, which were not related to AD pathology.

